# Flavonoid chrysin activates both TrkB and FGFR1 receptors while upregulates their endogenous ligands such as brain derived neurotrophic factor to promote human neurogenesis

**DOI:** 10.1111/cpr.13732

**Published:** 2024-09-27

**Authors:** Xiaoxu Dong, Gang Pei, Zhuo Yang, Shichao Huang

**Affiliations:** ^1^ School of Life Science and Technology Shanghai Tech University Shanghai China; ^2^ State Key Laboratory of Cell Biology, Shanghai Institute of Biochemistry and Cell Biology, Center for Excellence in Molecular Cell Science Chinese Academy of Sciences Shanghai China; ^3^ Shanghai Key Laboratory of Signaling and Disease Research, Laboratory of Receptor‐Based Biomedicine, The Collaborative Innovation Center for Brain Science, School of Life Sciences and Technology Tongji University Shanghai China; ^4^ Institute for Stem Cell and Regeneration Chinese Academy of Sciences Beijing China

## Abstract

Neurogenesis is the process of generating new neurons from neural stem cells (NSCs) and plays a crucial role in neurological diseases. The process involves a series of steps, including NSC proliferation, migration and differentiation, which are regulated by multiple pathways such as neurotrophic Trk and fibroblast growth factor receptors (FGFR) signalling. Despite the discovery of numerous compounds capable of modulating individual stages of neurogenesis, it remains challenging to identify an agent that can regulate multiple cellular processes of neurogenesis. Here, through screening of bioactive compounds in dietary functional foods, we identified a flavonoid chrysin that not only enhanced the human NSCs proliferation but also facilitated neuronal differentiation and neurite outgrowth. Further mechanistic study revealed the effect of chrysin was attenuated by inhibition of neurotrophic tropomyosin receptor kinase‐B (TrkB) receptor. Consistently, chrysin activated TrkB and downstream ERK1/2 and AKT. Intriguingly, we found that the effect of chrysin was also reduced by FGFR1 blockade. Moreover, extended treatment of chrysin enhanced levels of brain‐derived neurotrophic factor, as well as FGF1 and FGF8. Finally, chrysin was found to promote neurogenesis in human cerebral organoids by increasing the organoid expansion and folding, which was also mediated by TrkB and FGFR1 signalling. To conclude, our study indicates that activating both TrkB and FGFR1 signalling could be a promising avenue for therapeutic interventions in neurological diseases, and chrysin appears to be a potential candidate for the development of such treatments.

## Graphical abstract

In this study, we found a dietary flavonoid, chrysin, cooperatively activated tropomyosin receptor kinase‐B and FGFR1 signalling with up‐regulation of their endogenous ligands to promote human neurogenesis.

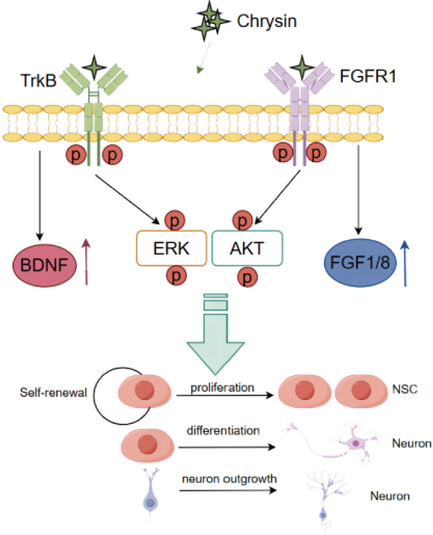

## 1 INTRODUCTION

Neurogenesis is the process by which new neurons are formed in the brain.^1^, ^2^ It occurs during embryonic development and continues in specific brain regions throughout an individual's lifespan.^3^ These newly generated neurons play a critical role in maintaining brain functions, including learning, memory and emotional regulation.^4^, ^5^ In human brains, neurogenesis mainly occurs in two regions: the subgranular zone of hippocampus and the subventricular zone of lateral ventricles.^6^, ^7^ These areas continuously generate neural stem cells (NSCs), leading to the generation of new neurons.^8^, ^9^, ^10^ The generation of neurons depends on precise coordination of intricate steps, including NSC proliferation, migration and differentiation.^11^ These processes are regulated by multiple signalling pathways, such as neurotrophic brain‐derived neurotrophic factor (BDNF)/tropomyosin receptor kinase‐B (TrkB) and fibroblast growth factor (FGF)/fibroblast growth factor receptor (FGFR) signalling.^2^, ^12^, ^13^ BDNF is a crucial neurotrophin responsible for neuronal survival, growth and differentiation.^14^, ^15^ Its high‐affinity receptor, TrkB, acts as a tyrosine kinase receptor.^16^ When activated by BDNF, TrkB initiates a cascade of intracellular signalling events that promote cell differentiation, survival, maturation and synaptic function.^17^ In addition to BDNF, the FGF family also plays essential roles in various developmental processes, including neurogenesis.^18^, ^19^, ^20^, ^21^ FGFs exert their effects by binding to specific cell surface receptors known as FGFRs.^22^ Activation of FGFRs triggers a cascade of intracellular signals that regulate cell proliferation, differentiation, migration and survival.^23^, ^24^, ^25^ Studies have shown that BDNF and TrkB receptor levels were found to decrease with age, suggesting that the signalling mechanisms responsible for maintaining neurogenesis may be impaired due to ageing.^26^, ^27^ Therefore, understanding the molecular mechanisms that modulate neurogenesis, and devising novel strategies that activate these neurotrophic signals in a cooperative manner, could be crucial in addressing cognitive decline associated with ageing and neurodegenerative diseases. However, the clinical use of neurotrophic endogenous ligands, such as BDNF, faces challenges due to their poor pharmacokinetics and potential side effects.^28^, ^29^ Hence, the search for agents that mimic neurotrophic factors has become a more promising avenue for maintaining neurogenesis.

Healthy lifestyle factors, especially a healthy diet, have been shown to positively influence neurogenesis.^30^, ^31^ This offers a promising avenue for exploring potential interventions for cognitive decline linked to ageing and neurodegenerative diseases. Flavonoids, a widespread category of polyphenols derived from plants, have been noted for their diverse biological and health‐enhancing properties.^32^, ^33^, ^34^, ^35^, ^36^ For example, quercetin, a flavonoid found naturally in many foods, is renowned for its anti‐ageing properties. It achieves this through multiple molecular mechanisms, such as modulating the SIRT1 pathway, exhibiting antioxidant and anti‐inflammatory activities, and enhancing autophagy.^37^ These findings suggest that flavonoids might be promising option for addressing cognitive decline related to ageing and neurodegenerative conditions by cooperatively modulating multiple pathways related to neurogenesis.

In the present study, we successfully identified a bioactive flavonoid, chrysin, promoted NSC proliferation and neuronal differentiation, which were both essential for neurogenesis. Mechanism study demonstrated that chrysin cooperatively active TrkB and FGFR1 receptor and upregulate expression of their endogenous ligands‐BDNF and FGF1/8. Moreover, the human organoid cerebral organoid model was used to test the effect of chrysin, and similar results were observed. Together, our findings indicate that activating both TrkB and FGFR1 signalling could be a promising avenue for therapeutic interventions in neurological diseases, and chrysin appears to be a potential candidate for the development of such treatments.

## 2 MATERIALS AND METHODS

### 2.1 Cell culture

Human induced pluripotent stem cell (iPSC)/iPSC‐derived NSCs (66‐year‐old healthy female [3 L]; 0‐year‐old female [13A]) were provided by IxCell Biotechnology, Ltd. The human induced pluripotent stem cells were cultured in Matrigel (Corning)‐coated dishes in mTeSR1 medium (Stem Cell Technology) under feeder‐free culture conditions. Accutase (Gibco) was used for enzymatic passaging. The colonies were divided into clumps every 6–7 days and then re‐plated on a dish coated with Matrigel. The medium was changed every day.

The human iPSC‐derived NSC cells were maintained as adherent culture in 50% Dulbecco's Modified Eagle Medium (DMEM)‐F12 and 50% neurobasal, containing 1× N2 supplement, 1× B27 supplement (Minus Vitamin A), 1× non‐essential amino acids (NEAA), 1× GlutaMAX, 10 ng/mL FGF‐Basic (AA10‐155) Recombinant Human Protein (basic FGF‐bFGF, Gibco), 10 ng/mL LIF recombinant Human Protein (hlif, Gibco), 3 μM CHIR99021 (Selleckchem), 5 μM SB431542 (Selleckchem) and 200 μM L‐ascorbic acid 2‐phosphate sesquimagnesium salt hydrate (Sigma).

All cells were cultured in a humidified incubator with 5% CO_2_/95% air (v/v) at 37°C.

### 2.2 Compounds and reagents

All compounds were purchased from MedChemExpress; the Cell Counting‐Lite 2.0 Luminescent Cell Viability assay was purchased from Vazyme. CCK‐8 Kit was purchased from Beyotime Biotechnology.

### 2.3 Plasmids and infection

As for RNA interference experiments, Short hairpin RNA (shRNAs) targeting human *TrkA*, *TrkB* and *FGFR1* were cloned in the PLKO.1 vector following the online protocol (Addgene, http://www.addgene.org/tools/protocols/plko/). All targeting sequences were listed in Table 1. The pLKO.1‐sh‐SCRAM vector expressing a disordered sequence that was not complementary to any human gene was used to be control.

**TABLE 1:** Primers for targeting sequences of quantitative PCR and shRNA used for gene silencing.

Genes	Primers (forward)	Primers (reverse)
*TrkA*	AACCTCACCATCGTGAAGAGT	TGAAGGAGAGATTCAGGCGAC
*TrkB*	TCGTGGCATTTCCGAGATTGG	TCGTCAGTTTGTTTCGGGTAAA
*Sox2*	ATGCACCGCTACGACGTGA	CTTTTGCACCCCTCCCATTT
*Nestin*	CAGCGTTGGAACAGAGGTTGG	TGGCACAGGTGTCTCAAGGGTAG
*DCX*	TCCCGGATGAATGGGTTGC	GCGTACACAATCCCCTTGAAGT
*Tubb3*	GGCCAAGGGTCACTACACG	GCAGTCGCAGTTTTCACACTC
*BDNF*	ACCTGAACACTTATTGCTTTG	CATTCGGCCTGAGTTTGG
*NGF*	GGCAGACCCGCAACATTACT	CACCACCGACCTCGAAGTC
*FGF1*	CTCCCGAAGGATTAAACGACG	GTCAGTGCTGCCTGAATGCT
*FGF2*	AGAAGAGCGACCCTCACATCA	CGGTTAGCACACACTCCTTTG
*FGF3*‐	GGCGTCTACGAGCACCTTG	CCACTGCCGTTATCTCCAAAA
*FGF4*	CTCGCCCTTCTTCACCGATG	GTAGGACTCGTAGGCGTTGTA
*FGF8*	GACCCCTTCGCAAAGCTCAT	CCGTTGCTCTTGGCGATCA
*EGF*	TGGATGTGCTTGATAAGCGG	ACCATGTCCTTTCCAGTGTGT
*GAPDH*	GGAGCGAGATCCCTCCAAAAT	GGCTGTTGTCATACTTCTCATGG
*TrkA*‐shRNA	CCGGTATCTACAGCACCGACTATTACTCGAGTAATAGTCGGTGCTGTAGATATTTTTG	AATTCAAAAATATCTACAGCACCGACTATTACTCGAGTAATAGTCGGTGCTGTAGATA
*TrkB*‐shRNA	CCGGCCTTAAGGATAACTAACATTTCTCGAGAAATGTTAGTTATCCTTAAGGTTTTTG	AATTCAAAAACCTTAAGGATAACTAACATTTCTCGAGAAATGTTAGTTATCCTTAAGG
*FGFR1*‐shRNA	CCGGCCACTCCTCAGTCGCTATATTCTCGAGAATATAGCGACTGAGGAGTGGTTTTTG	AATTCAAAAACCACTCCTCAGTCGCTATATTCTCGAGAATATAGCGACTGAGGAGTGG

For virus packaging and knockdown experiments were similar to previously reported with minor modification.^38^ In brief, human NSCs were seeded in 60‐mm dishes, then were infected with virus in the presence of polybrene (Sigma, 4 μg/mL). After 8 h, the medium was refreshed. The efficiency of the knockdown was determined by quantitative real‐time polymerase chain reaction (qRT‐PCR) or Western blot at 72 or 96 h post‐infection.

### 2.4 Cell titre glo assay

Cell growth was detected by using the Cell Counting‐Lite 2.0 Luminescent Cell Viability assay. Firstly, human NSCs were plated in the 96‐well plates with a density of 3500 cells per well and cultured in medium containing 1 ng/mL basic fibroblast growth factor (bFGF). After 24 h, the treatment of compounds was performed and sustained for 48/72 h. Luminescence was recorded using a multifunctional microporous detector (BioTek SynergyNEO).

### 2.5 Cell counting kit 8 assay

Cell proliferation experiments were also detected using Cell Counting Kit 8 (CCK8) assay. Human NSCs were seeded in the 96‐well plates with 3500 per well and detected after 48 h treatment. Then the solution of CCK8 (10 μL) was added to each well and incubated at 37°C for 2 h. Finally, the absorbance was measured at 450 nm with a microplate spectrophotometer (BioTek SynergyNEO).

### 2.6 EdU assay

5‐Ethynyl‐2'‐deoxyuridine (EdU) kit (Beyotime, C0075L) was used for the EdU cell proliferation assay. Briefly, cells were treated with compounds for 48 h, then cultured with 1:1000 diluted EdU for 2 h. After that, the cells were fixed with 4% paraformaldehyde and stained with fluorescent dye and 4',6‐Diamidino‐2‐phenylindole (DAPI).

### 2.7 Immunofluorescence and imaging

In order to facilitate the experiment, cells were seeded in the 24 well plated with 1 × 1 cm slides. After 48 h treatment of compounds, the cells were fixed with 4% paraformaldehyde for 15 min. And then gently washed the cells with 1× PBS for three times. After that, 0.3% TritonX‐100 was used for permeabilized the cells. Next, the bovine serum albumin was used to block the samples for 1 h. The primary antibodies included Sox2 (R&D), Ki67 (Abcam), DCX (Santa Cruz Technology) and Nestin (Thermo Fisher Technology), which were incubated overnight at 4°C. After day, samples were washed by 1× PBS, then incubated with species‐specific secondary antibody for 1 h at real‐time (RT). DAPI was used to visualise the nuclei for 15 min. Lastly, the slides were sealed and dried for the imaging. All images were obtained by laser scanning confocal microscope (Olympus, FV3000). A list of all antibodies with catalogue numbers is attached in Table 2.

**TABLE 2:** Resource of antibodies.

Antibody	Source	Identifier
Rabbit anti TrkB	CST	4603
Rabbit anti p‐TrkB(Y816)	CST	4168S
Rabbit anti p‐TrkB(Y516)	CST	4619T
Rabbit anti p‐TrkB(Y707)	CST	4621T
Rabbit anti FGFR1	CST	9740S
Rabbit anti p‐FGFR1	CST	Ab173305
Rabbit anti ERK1/2	CST	4695S
Rabbit anti p‐ERK1/2	CST	4370S
Rabbit anti AKT	ABclonal	A17909
Rabbit anti p‐AKT	ABclonal	AP1208
Rabbit anti BDNF	ABclonal	A16299
Rabbit anti FGF1	ABclonal	A23167
Rabbit anti FGF2	ABclonal	A22330
Rabbit anti actin	Beyotime Biotechnology	AF5003
Goat anti Sox2	R&D	AF2018
Mouse anti Nestin	Thermo Fisher Technology	PA5‐11887
Mouse anti DCX	Santa Cruz	SC‐271390
Rabbit anti Ki67	Abcam	ab15580

Abbreviations: BDNF, brain‐derived neurotrophic factor; CST, Cell Signalling Technology; TrkB, tropomyosin receptor kinase‐B.

### 2.8 Neurosphere assay

The NSCs were seeded into six‐well clear flat bottom ultra low attachment plates (Corning) with a density of 2 × 10^4^ per well. The growth medium was composed of DMEM/F12 (Gibco), B27 supplement (Gibco), 20 ng/mL bFGF (Gibco), 20 ng/mL epidermal growth factor (EGF) (STEMCELL Technologies) and heparin (STEMCELL Technologies). The next day, the cells were treated with compounds. Then all cells were cultured for 7 days to form primary neurospheres. On Day 7, the primary neurospheres were dissociated and plated in the 96‐well clear flat bottom ultra‐low attachment plates at the density of 500 per well and cultured for 7 days to form secondary neurospheres. Furthermore, the medium with compounds was supplemented on Day 4 and Day 11. And the inhibitors were pretreated before compounds treatments for 1 h.

### 2.9 Human neural stem cell differentiation and Sholl analysis

For neuronal differentiation, human NSCs were seeded in 24‐well plates coated by poly‐D‐lysine and laminin with a density of 5 × 10^4^ cells per well. After a day, the medium was changed to neuron differentiation medium comprised of neurobasal basal medium with 1× B27, 1× Culture One Supplement (Thermo Fisher Scientific), 1× GlutaMAX and 200 μM ascorbic acid. The differential medium was refreshed every other day. On Day 10 of differentiation, cells were treated with compounds for 72 h.

After treatment by compounds, the samples were fixed with 4% paraformaldehyde and then immunofluorescence with neuron marker‐Tuj1. Next, the images were gained by a confocal microscope (Olympus, FV3000). The neuroanatomy plugins of Image J were used for Sholl analysis. In brief, concentric circles in 20 μm radius increments were superimposed around the canter of the soma, and the number of dendrites crossing each circle was quantified in a blinded manner.

### 2.10 Neurite length analysis

SHSY‐5Y cell were used for the neurite length detection. Firstly, cells seeded in 12‐well plates with MEM/F12 medium containing 10% fetal bovine serum. Next day, cells were treated with compounds for 24 h. Then imaging was performed by an inverted microscope (Olympus, IX73). Ten visual fields were randomly shot in each hole, and the longest five neurites in each visual field were selected for length statistics.

### 2.11 RNA extraction and qRT‐PCR

RNA extraction was performed with EZ‐press RNA Purification Kit (EZB, B0004D). Then reverse transcription was performed with 5X *Evo M‐MLV* RT Master Mix (Accurate Biology, AG11706). All gene transcripts were quantified by qRT‐PCR performed with a 2× HotStart SYBR Green qPCR Master Mix (ExCell Bio) on a Stratagene Mx3000P (Agilent Technologies). The primers used for the detection of mRNA levels of human genes are listed in Table 1.

### 2.12 Western blot

To detect TrkB and FGFR1 activation, the cells were seeded in 12‐well plates and treated for 10 min with indicated compounds. Then the samples were lysed on ice with 1× sodium dodecyl sulfate (SDS) loading. Equal quantity of proteins per lane was separated by 10% sodium dodecyl sulfate polyacrylamide gel electrophoresis (SDS–PAGE) and transferred onto nitrocellulose membranes (Cytlva). Then membranes were blocked by 5% milk for 1 h and incubated with primary antibodies including total and phosphorylated TrkB/FGFR1 and their downstream effectors—ERK and AKT (Cell Signalling Technology, CST) at 4°C overnight. The membranes were washed with tris‐buffered saline with tween buffer for three times. Then the membranes were processed with consistent species secondary antibodies for 1 h. At last, the targeted proteins were visualised with enhancer luminous fluid (Bio Rad) on the Chemiluminescence Imager.

For growth factors expression detection, the cells in 12‐well plates were treated for 24 h. Then bicinchoninic acid (Thermo Fisher Scientific) was used for confirming the concentration of proteins. After that, equal quantity of proteins per lane was separated by 12.5% SDS–PAGE. Then the next steps were similar to those above. And all primary antibodies used in this assay were from ABclonal Technology.

The quantification of Western blot bands was performed as follows. Individual files were loaded in ImageJ software, and the intensity of the Western blotting bands was determined by measuring the grey values. In order to obtain the intensities of different bands, a rectangular region of interest (ROI) was made to cover the area of one band, and the same ROI was used for all bands. Integrated grey values of these bands were obtained using the gel analyses tools in ImageJ. The data from four independent experiments were analysed by prism 9.5. A list of all antibodies used in Western blot with catalogue numbers is attached in Table 2.

### 2.13 Human cerebral organoids culture

Consisted with before, Human Cerebral Organoids Culture was performed with STEMdiff Cerebral Organoid Kit (STEMCELL Technologies). Briefly, human iPSCs were seeded in the 96‐well U‐shaped plates (Corning) with 1 × 10^4^ per well and then centrifuged at 1000 × *g* for 5 min to form single embryoid bodies. After a day, EB medium was changed. Then the medium was changed every 2 days. On Day 5, half of the medium was replaced with induction medium. On Day 7, organoids were implanted into Matrigel (Corning) and continued to grow in the expansion medium with 12‐well plates (Corning) in suspension culture. At the same time, the compounds treatment started. After 1 h of treatment with inhibitors, the vehicle (dimethyl sulfoxide) and chrysin (10 μM) were added to each group. Three days later, the implanted organoids were transferred into mature medium (supplemented with inhibitors and chrysin) and cultured on a shaker until the end of the experiment. The inhibitors and chrysin were supplemented while changing the medium every 3 days. One week after compound treatment, the analysis of organoid morphology was performed by light‐sheet fluorescence microscopy. Immunofluorescence analysis of multiple markers was performed 2 weeks after compound treatment.

## 3 RESULTS

### 3.1 Chrysin enhanced the proliferation and self‐renewal of human NSCs

**FIGURE 1:**
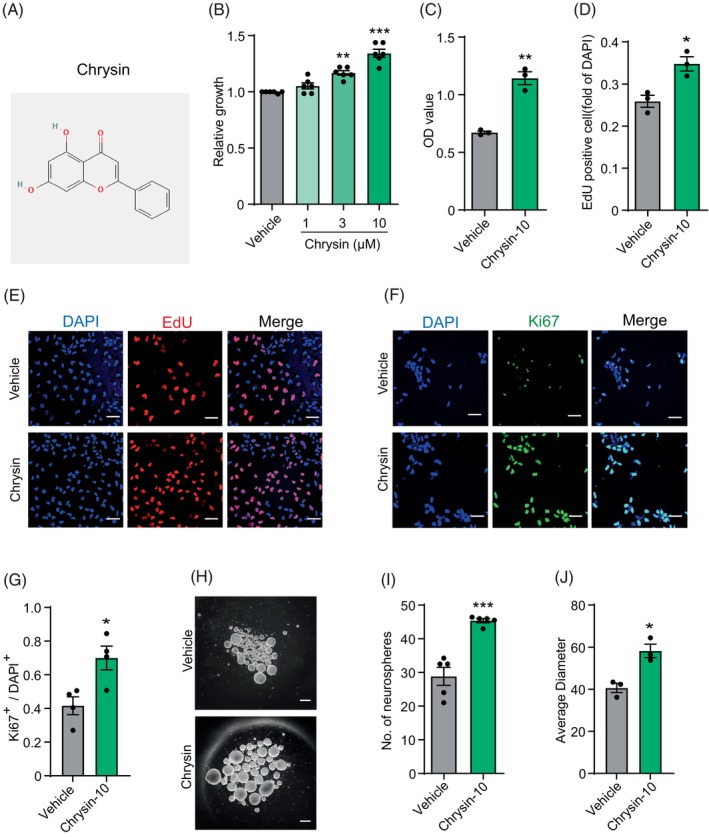
Chrysin enhanced the proliferation and self‐renewal of neural stem cell. (A) The structure of chrysin. (B) Dosage‐dependent response of cell growth after treatment with chrysin in the presence of low bFGF concentration determined by cell titre glo. Data are mean ± SEM. **p < 0.005, ***p < 0.001. One‐way ANOVA, followed by Tukey's multiple comparisons test. *N* = 6. (C) The effect of chrysin on human neural stem cells (NSC) growth in the presence of low bFGF concentration determined by CCK8 assay. Data are mean ± SEM. **p < 0.005, unpaired Student's *t*‐test. *N* = 3. (D) The percentage of EdU‐positive cells to the control after treatment with chrysin. Data are mean ± SEM. *p < 0.05, unpaired Student's *t*‐test. *N* = 3. (E) Representative images of EdU immunostaining. Scale bar = 100 μm. (F) Immunostaining of Ki67/DAPI after chrysin treatment. Scale bar = 100 μm. (G) Quantification of the Ki67 positive cells between control and chrysin treatment groups. Data are mean ± SEM. *p < 0.05, unpaired Student's *t*‐test. *N* = 4. (H) Representive images of secondary neurospheres formed from human NSC with or without chrysin treatment. Scale bar = 100 μm. (I, J) The number and the diameters of secondary neurospheres after chrysin treatment. Data are mean ± SEM. *p < 0.05, ***p < 0.001, unpaired Student's *t*‐test. *N* = 5, *N* = 3. ANOVA, analysis of variance; DAPI: 4',6‐Diamidino‐2‐phenylindole; EdU, 5‐Ethynyl‐2'‐deoxyuridine; SEM, standard error of the mean.

Precise regulation of NSCs proliferation and neuron growth is crucial for the development and function of the human brain. To identify bioactive compounds in dietary functional foods that promote both NSC proliferation and neuronal growth, we sequentially employed two assays: cell growth assay and neurite outgrowth assay. The results indicated that, among the 20 natural flavonoids tested, eight significantly promoted the proliferation of NSCs (Figure S1A). Then SHSY‐5Y cells were treated with the eight flavonoids to detect the neurite length. At the same time, retinoic acid (RA), a potent neuritogenic drug for SHSY‐5Y cells, was used as a positive control. We found that chrysin showed strong effects on neurite outgrowth (Figure S1B). Therefore, we focused on chrysin in subsequent studies. The structure of chrysin is shown (Figure 1A). The result showed that chrysin dose‐dependently increased the proliferation of human NSCs, with the maximum effect observed at a concentration of 10 μM (Figure 1B). Furthermore, the proliferative response was also observed to intensify over time (Figure S1C). To substantiate these effects, another NSC cell line was utilised to validate the impact of chrysin on cell proliferation. Subsequently, CCK 8 assay and EdU incorporation assay were performed to verify the effects of chrysin on NSC proliferation (Figure 1C–E). We also conducted immunofluorescence staining to detect the proliferative marker Ki‐67 (Figure 1F,G). The above results collectively demonstrated that chrysin significantly enhanced the proliferation of human NSCs. Besides, the effects of chrysin on NSCs self‐renew were evaluated with a neurosphere assay. The result indicated that chrysin treatment not only increased the number of neurospheres, but also enlarged their size (Figures 1H–J and S1E).

### 3.2 Chrysin promoted NSCs differentiation and neuron outgrowth

**FIGURE 2:**
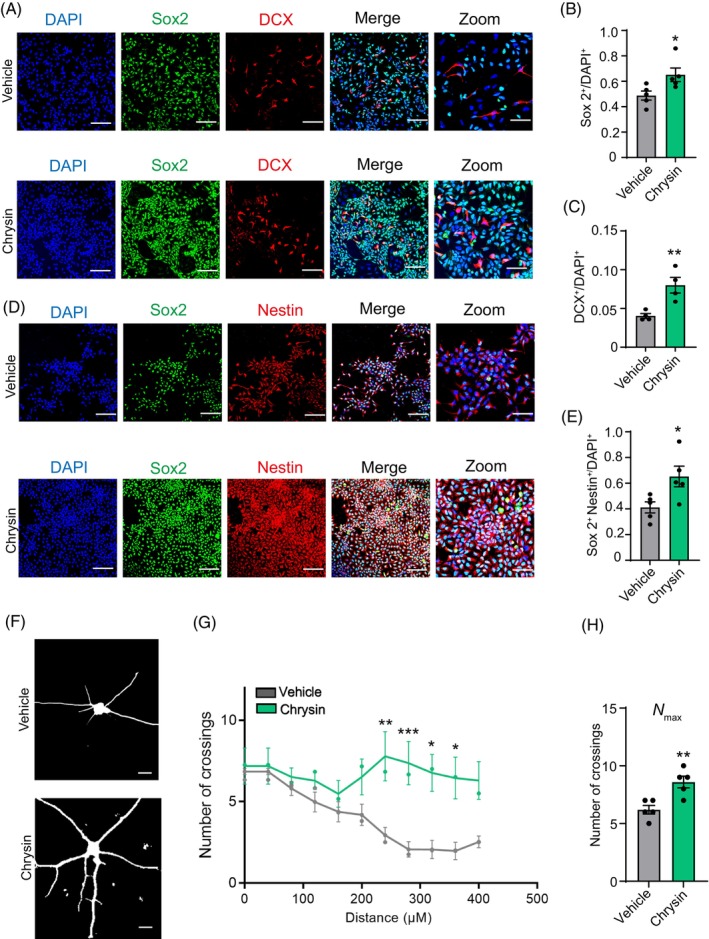
Chrysin promoted neuron differentiation and outgrowth. (A) Immunostaining of the Sox2/DCX in vehicle‐ or chrysin‐treated human neural stem cells (NSCs). Scale bar = 200 μm. (B) Quantification of the Sox2^+^ cells in human NSCs with or without chrysin treatment. Data are mean ± SEM. *p < 0.05, unpaired Student's *t*‐test. *N* = 5. (C) Quantification of the DCX^+^ cells in human NSCs with or without chrysin treatment. Data are mean ± SEM. **p < 0.005, unpaired Student's *t*‐test. *N* = 4. (D) Representative images of immunostaining for Sox2^+^/Nestin^+^ in vehicle‐ or chrysin‐treated human NSCs. Scale bar = 200 μm. (E) Quantification of the co‐immunostaining of Sox2^+^ and Nestin^+^ cells in human NSCs with or without chrysin treatment. Data are mean ± SEM. *p < 0.05, unpaired Student's *t*‐test. *N* = 5. (F) Representive images of NSC‐derived neuron with or without chrysin treatment. Scale bar = 100 μm. (G) Sholl analysis demonstrates dendritic arbour complexity in NSC‐derived neuron after chrysin treatment. *N* = 5 (*n* = 4 neuron per condition). Data are mean ± SEM. *p < 0.05, **p < 0.005, ***p < 0.001, One‐way ANOVA, followed by Tukey's multiple comparisons test. (H) Maximum number of crossings (*N*_max_) of the Sholl plots in (G). Data are mean ± SEM. **p < 0.005, *N* = 5. ANOVA, analysis of variance; SEM, standard error of the mean.

Next, we further assessed the effect of chrysin on NSCs differentiation and neurite outgrowth. We analysed the mRNA expression levels of marker genes associated with NSCs (*Sox2* and *Nestin*) and neurons (*DCX* and *Tuj1*). Compared with the control group, chrysin treatment upregulated the mRNA expression of all these genes (Figure S2A–D). Immunofluorescence staining results showed that treatment of chrysin increased the ratio of *Sox2* ^+^ and *SOX2* ^+^ *Nestein* ^+^ NSCs, as well as an increase in *DCX* ^+^ immature neurons in comparison to the control group (Figure 2A–E). These results imply that chrysin not only promotes the proliferation of NSCs but also facilitates their differentiation into neurons. Then we found that the ratio of GFAP+/Sox2^−^ astrocytes was decreased after chrysin treatment (Figure S2E,F). To further verify the influence of chrysin on astrocytic differentiation, an astroglia differentiation protocol was also performed, the results demonstrated that treatment with chrysin significantly influenced the process. The ratio of GFAP^+^Sox2^−^/DAPI^+^ reduced compared with control (Figure S2G,H). Collectively, our results show that chrysin could enhance the neural differentiation of the human NSCs but inhibited the differentiation to astrocytes. We next evaluated the neurite outgrowth of SHSY‐5Y cells following treatment with chrysin, using RA as a positive control. The result indicated that chrysin significantly enhanced neurite outgrowth (Figure S2I–K). To further validate these results, we used neurons derived from NSCs. Following chrysin treatment, we performed immunofluorescence staining for *TUJ1*. Subsequent analysis of the images using Sholl analysis revealed that chrysin increased dendritic arbour complexity, which was measured by both the maximum number of crossings and the area under the curve of the Sholl plots (Figure 2F–H). These results suggested that chrysin significantly promoted neurite outgrowth.

### 3.3 Neurotrophin receptor TrkB with its endogenous ligand BDNF mediated the function of chrysin

**FIGURE 3:**
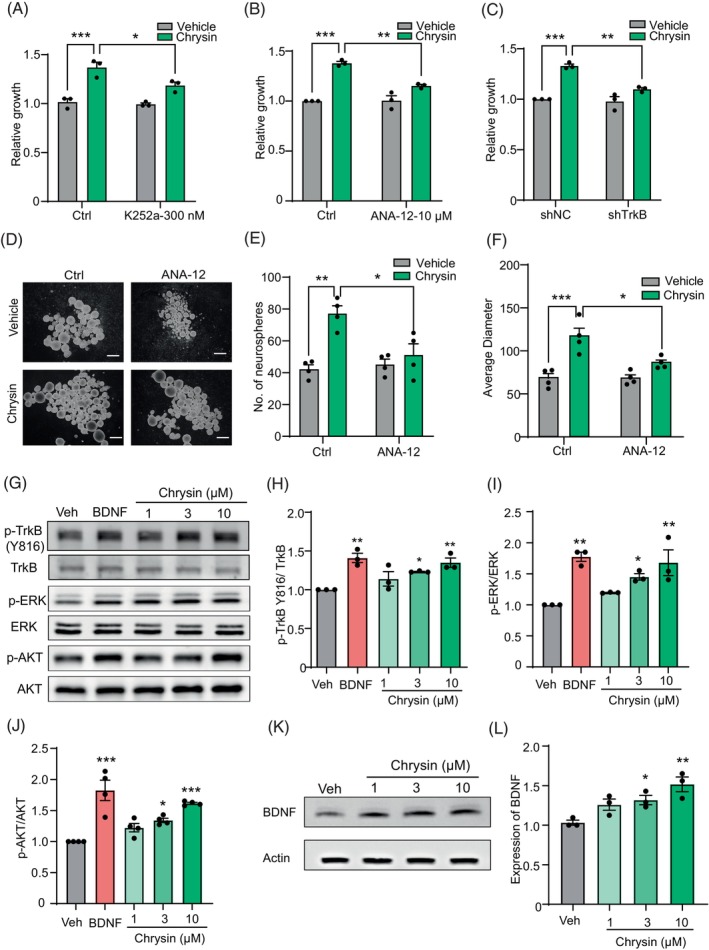
Neurotrophin receptor TrkB with its endogenous ligand BDNF mediated the function of chrysin. (A) The effects of chrysin on neural stem cells (NSC) proliferation with pan‐inhibitor (K252a) of Trks. Data are mean ± SEM. *p < 0.05, ***p < 0.001, one‐way ANOVA, followed by Tukey's multiple comparisons test. *N* = 3. (B) The effect of chrysin on human NSC growth with TrkB inhibitor (ANA‐12) pretreatment. Data are mean ± SEM. **p < 0.01, ***p < 0.001, one‐way ANOVA, followed by Tukey's multiple comparisons test. *N* = 3. (C) The effect of chrysin on human NSC growth with TrkB knock down. Data are mean ± SEM. **p < 0.01, ***p < 0.001, one‐way ANOVA, followed by Tukey's multiple comparisons test. *N* = 3. (D–F) The formation of secondary neurospheres after chrysin treatment with TrkB inhibitor pretreatment. Data are mean ± SEM. *p < 0.05, **p < 0.01, ***p < 0.001, one‐way ANOVA, followed by Tukey's multiple comparisons test. *N* = 4. (G) The p‐TrkB/p‐ERK/p‐AKT protein levels in human NSC after treatment with chrysin. (H–J) Statistic of the ratio of phosphorylated protein to total protein in (G). Data are mean ± SEM. *p < 0.05, **p < 0.01, ***p < 0.001, one‐way ANOVA, followed by Tukey's multiple comparisons test. *N* = 3. (K) The expression of BDNF with chrysin treatment in human NSCs. (L) Statistic of the protein levels of BDNF. *p < 0.05, **p < 0.01, one‐way ANOVA, followed by Tukey's multiple comparisons test. *N* = 3. SEM, standard error of the mean.

Given that the flavonoid chrysin promotes neurogenesis in human NSC, we next explored the molecular mechanisms underlying chrysin's effects. Since neurotrophin receptors‐mediated signalling pathways play a key role in regulating the fate of NSCs, we hypothesise that chrysin's effects on NSCs are also mediated by neurotrophin receptors. We first assessed the expression levels of three neurotrophin receptors, and the results indicated that TrkB was the most abundant receptor compared to TrkA and TrkC in human NSCs (Figure S3A). Then we investigated whether the effect of chrysin could be blocked by the inhibitor of Trks. The results demonstrated that inhibitor (K252a) targeting Trk receptors diminished the effects of chrysin on NSC proliferation (Figure 3A). After that, the selective inhibitors of TrkA and TrkB were used to ensure the regulation receptors. We found inhibition of TrkB obviously decreased the effects of chrysin on NSC proliferation rather than TrkA (Figures 3B and S3B). Afterwards, knockdown of TrkB and TrkA obtained similar results with inhibitors (Figures 3C and S3E). Identification of knockdown efficiency was ensured by qRT‐PCR (Figure S3C,D). Overall, we demonstrated that TrkB mediated the effects of chrysin on NSC proliferation.

Next, we analysed the impacts of the TrkB inhibition on NSC self‐renewal. The results are consistent with NSC proliferation assay that inhibition of TrkB reduced the effects of chrysin on NSC self‐renewal (Figure 3D–F). Our data to this point demonstrate a critical role for TrkB receptor in chrysin‐mediated neurogenesis in vitro. Additionally, we detected the response of TrkB after chrysin treatment with different concentrations and time points by Western blot in NSCs. Endogenous ligand of TrkB–BDNF was used as the positive control. The results indicated that chrysin could upregulate TrkB phosphorylation as well as the downstream factors—ERK1/2 and AKT (Figures 3G–J and S3F). In addition, to further explore the interactions between chrysin and TrkB, molecular docking was performed using Maestro and the results revealed that chrysin has a docking score of −5.107 and forms hydrogen bonds with ASP298 and THR296, Pi‐Pi Stacked with HIS343 (Figure S3G).

Up‐regulation of neurotrophins also could promote the process of neurogenesis. Hence, we continue to detect the expression of neurotrophins; the results indicated treatment with chrysin significantly increased expression of BDNF not only at the mRNA level but also at protein levels with nerve growth factor as control (Figures S3H,I and 3K,L).

### 3.4 FGF/FGFR1 signalling also mediated the effects of chrysin

**FIGURE 4:**
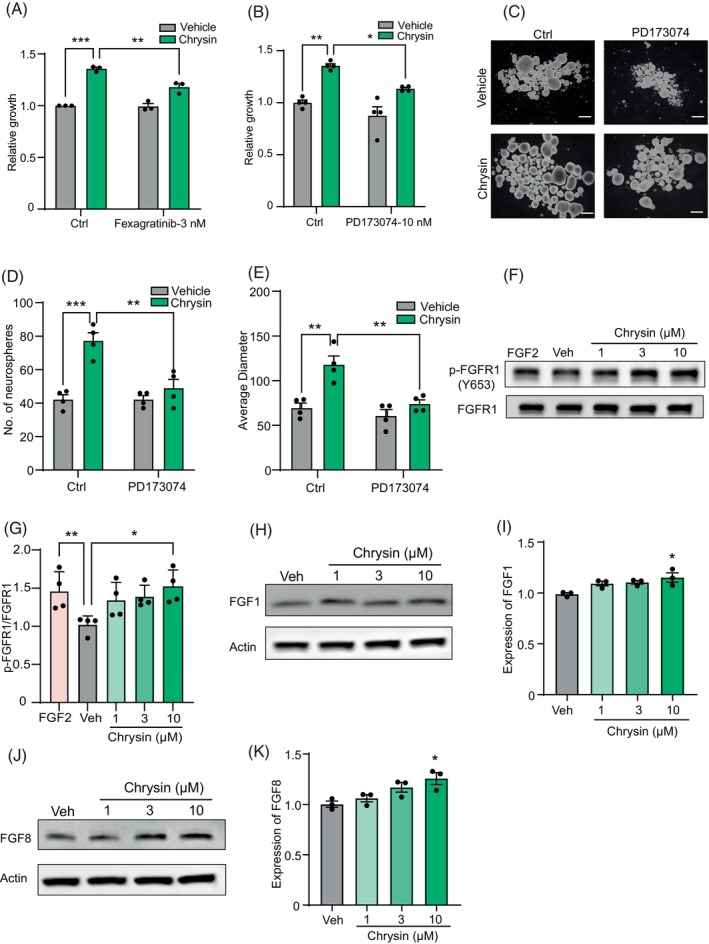
Fibroblast growth factor (FGF)/FGFR1 signalling also mediated the effects of chrysin. (A) The effects of chrysin on neural stem cells (NSC) proliferation with pan‐inhibitor (Fexagratinib) of fibroblast growth factor receptors (FGFRs). Data are mean ± SEM. **p < 0.01, ***p < 0.001, one‐way ANOVA, followed by Tukey's multiple comparisons test. *N* = 3. (B) The effect of chrysin on human NSCs growth in the presence or absence of FGFR1 inhibitor (PD173074) pretreatment. Data are mean ± SEM. *p < 0.05, **p < 0.01, one‐way ANOVA, followed by Tukey's multiple comparisons test. *N* = 4. (C–E) Representative images and quantifications of secondary neurospheres with chrysin treatment in the presence or absence of FGFR1 inhibitor pretreatment. Data are mean ± SEM. **p < 0.01, ***p < 0.001, one‐way ANOVA, followed by Tukey's multiple comparisons test. *N* = 4. (F) The p‐FGFR1 protein levels in human NSC after treatment with chrysin. (G) The ratio of phosphorylated (Y653) to total FGFR1 protein. Data are mean ± SEM. *p < 0.05, **p < 0.01, one‐way ANOVA, followed by Tukey's multiple comparisons test. *N* = 4. (H) The expression of FGF1 with chrysin treatment in human NSCs. (I) Statistic of the protein levels of FGF1. *p < 0.05, one‐way ANOVA, followed by Tukey's multiple comparisons test. *N* = 3. (J) The expression of FGF8 with chrysin treatment in human NSCs. (I) Statistic of the protein levels of FGF8. *p < 0.05, one‐way ANOVA, followed by Tukey's multiple comparisons test. *N* = 3.

Given the effects of chrysin with TrkB inhibition, we hypothesised there are other modulators involved in the regulation of chrysin. Hence, we performed the inhibition of receptor tyrosine kinases involved in the regulation of neurogenesis. The results demonstrated that inhibitor (Fexagratinib) targeting FGFRs reduced the effects of chrysin on NSC proliferation, whereas inhibitors targeting epidermal growth factor receptor and VEGFR2 did not exhibit significant impact (Figures 4A and S4A,B). Then we detected the abundance of four FGFRs and results showed FGFR1 was the highest subtype that indicated its important role in human NSCs (Figure S4C). Therefore, the selective inhibitor (PD173074) of FGFR1 was pretreated with chrysin. Notably, inhibition of FGFR1 significantly reduced the effects of chrysin on NSC proliferation (Figure 4B) Consistently, the similar effects were found in the self‐renewal assay (Figure 4C–E). Then, we next investigated the effect of chrysin on FGFR1 activation. The experiment showed that chrysin also could activate FGFR1 (Figure 4F,G). Similar to above, molecular docking was performed using Maestro for detection of interaction between chrysin and FGFR. We found that chrysin has a docking score of −4.578 and formed hydrogen bonds with LYS163, LYS175 and THR173 (Figure S4D). Next, we continue to detect the growth factors expression with EGF as the control. Results of mRNA levels indicated that treatment of chrysin significantly increased expression of FGF1 and FGF8 (Figure S4E–J). Then we proceed to examine the expression of the above three factors by Western blot. Our results corroborated with the mRNA levels that treatment of chrysin obviously upregulated the expression of FGF1 and FGF8 (Figure 4H–K).

Overall, our data revealed that chrysin cooperatively activates TrkB and FGFR1 with the downstream factors to promote human neurogenesis. Further, in order to study the relationship between TrkB and FGFR1, we co‐treated NSCs with the two inhibitors before chrysin treatment and found that the increase in proliferation induced by chrysin was almost abolished (Figure S4K). Analogous results were obtained in the neurosphere assay (Figure S4L–N). However, the results on neurite outgrowth indicated a bit of a difference: inhibitors of TrkB and FGFR1 solely decreased the effects of chrysin, but co‐treated of the two inhibitors did not exhibit superposition effects (Figure S4O).

### 3.5 Chrysin promote neurogenesis in human cerebral organoid

**FIGURE 5:**
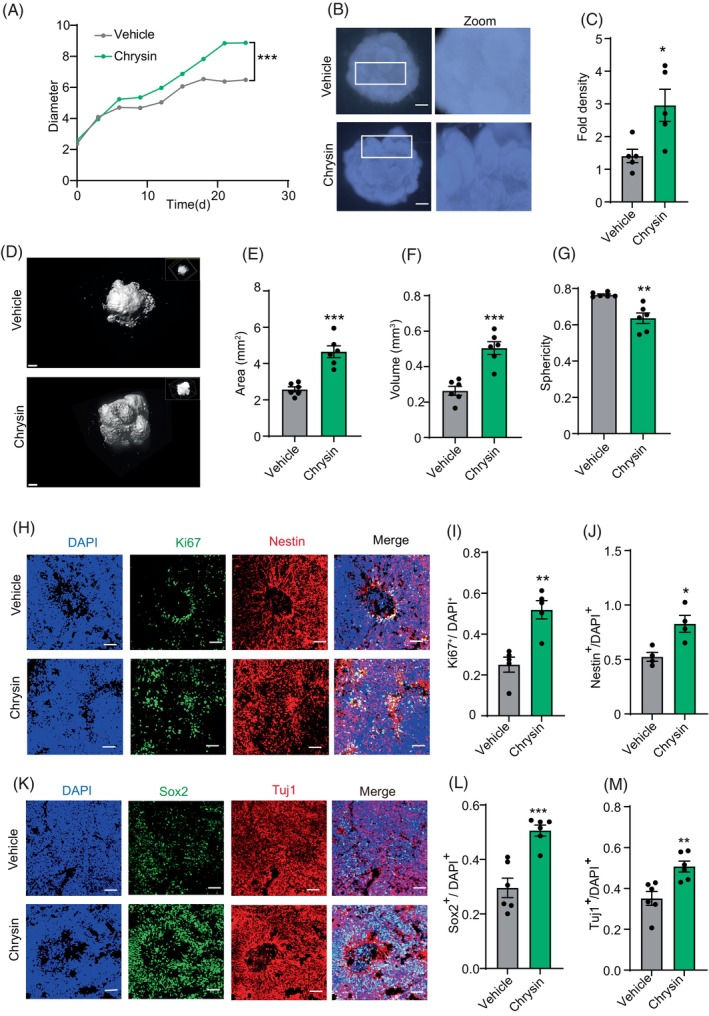
Chrysin promoted neurogenesis in human cerebral organoids. (A) The diametres curve of cerebral organoids with chrysin treatment. Data are mean ± SEM. ***p < 0.001, one‐way ANOVA, followed by Tukey's multiple comparisons test. *N* = 3. (B, C) Higher magnification view of the top of control and chrysin‐treated organoid using the stereological method and quantification of surface folds. Scale bar = 100 μm. Data are mean ± SEM. *p < 0.05, unpaired Student's *t*‐test. *N* = 5. (D–G) Light‐sheet images and reconstructed models of control and chrysin‐treated organoids and quantification of volume, surface area, and sphericity at 2 weeks (*n* = 3 cerebral organoids per condition). Scale bar = 200 μm. Data are mean ± SEM. **p < 0.01, ***p < 0.001, unpaired Student's *t*‐test. *N* = 6. (H) Representative images of Ki67^+^ and Nestin^+^ cells in control or chrysin‐treatment cerebral organoids at 3 weeks. Scale bar = 200 μm. (I) Quantification of Ki67^+^ cells to DAPI^+^ cells with or without chrysin treatment. Data are mean ± SEM. **p < 0.01, unpaired Student's *t*‐test. *N* = 5. (J) Quantification of Nestin^+^ cells to DAPI^+^ cells with or without chrysin treatment. Data are mean ± SEM. *p < 0.05, unpaired Student's *t*‐test. *N* = 4. (K) Immunostaining of Sox2^+^ and Tuj1^+^ cells in control or chrysin‐treated cerebral organoids at 3 weeks. Scale bar = 200 μm. (L, M) Quantification of Sox2^+^ and Tuj1^+^ cells to DAPI^+^ cells with or without chrysin treatment. Data are mean ± SEM. **p < 0.01, ***p < 0.001, unpaired Student's *t*‐test. *N* = 6.

To explore the impact of chrysin on neurogenesis within a model akin to the human brain, we utilised human cerebral organoids that emulate the cellular and architectural characteristics of human brain tissue. We treated the organoids with chrysin starting from Day 7 of organoid formation and measured their diameters every 3 days until 3 weeks after the chrysin treatment. After 5 days of treatment, we observed that the cerebral organoids in the chrysin treatment group began to exhibit a longer diameter compared to the control group, continuing until the end of the experiments (Figure 5A). At the same time, we found that with cerebral organoid development, chrysin‐treated group displayed more ventricle‐like structures and exhibited markedly increased folding. We applied to the folding degree by stereoscope and confirmed the increased folding after chrysin treatment (Figure 5B,C). Furthermore, we employed a light‐sheet fluorescence microscope to capture images of the cerebral organoid after 3 weeks of treatment, followed by 3D structure remodelling using Imaris (Figure 5D). The result demonstrated that the chrysin‐treated group exhibited increased area and volume, along with a decrease in sphericity (Figure 5E,F). In summary, our results demonstrated chrysin treatment promoted the expansion and folding of cerebral organoid.

To investigate whether the increase of cerebral organoid expansion and folding after chrysin treatment was linked to alterations in neurogenesis, we performed immunofluorescent staining on these cerebral organoids. In line with the increased organoid expansion after chrysin treatment, we observed more Ki67^+^ proliferating cells in chrysin‐treated cerebral organoids (Figure 5H,I). Additionally, chrysin‐treated group also exhibited higher levels of Sox2^+^ and Nestin^+^ NSCs (Figure 5H–L). Moreover, organoids from the chrysin‐treated group exhibited an enhanced presence of Tuj1‐positive neurons compared to organoids from the control group, indicating that chrysin also promotes neuronal differentiation (Figure 5K,M). Overall, our results in cerebral organoids confirm the role of chrysin in promoting neurogenesis.

### 3.6 Blockage of TrkB and FGFR1 reduced effects of chrysin on cerebral organoids

**FIGURE 6:**
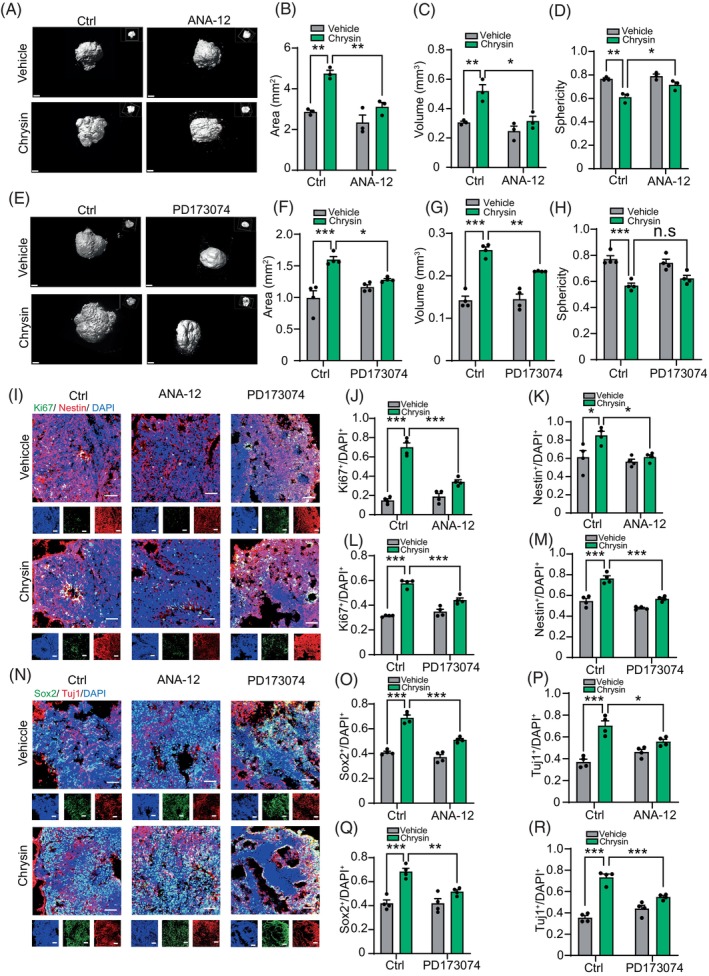
Blockage of tropomyosin receptor kinase‐B (TrkB) and FGFR1 reduced the effects of chrysin in cerebral organoids. (A–D) Light‐sheet images and reconstructed models of control and chrysin‐treated organoids with or without TrkB inhibitor (ANA‐12) pretreatment and quantification of volume, surface area, and sphericity at 2 weeks (*n* = 3 cerebral organoids per condition). Scale bar = 200 μm. Data are mean ± SEM. *p < 0.05, **p < 0.01, one‐way ANOVA, followed by Tukey's multiple comparisons test. *N* = 3. (E–H) Light‐sheet images and reconstructed models of control and chrysin‐treated organoids with or without FGFR1 inhibitor (PD173074) pretreatment and quantification of volume, surface area and sphericity at 2 weeks (*n* = 3 cerebral organoids per condition). Scale bar = 200 μm. Data are mean ± SEM. *p < 0.05, **p < 0.01, ***p < 0.001, one‐way ANOVA, followed by Tukey's multiple comparisons test. *N* = 3. (I) Immunostaining of Ki67^+^ and Nestin^+^ cells in cerebral organoids in presence or absence of TrkB and FGFR1 inhibitors pretreatment. Scale bar = 200 μm. (J, K) The ratio of Ki67^+^ and Nestin^+^ in cerebral organoids with TrkB inhibitor (ANA‐12) pretreatment. Data are mean ± SEM. *p < 0.05, ***p < 0.001, one‐way ANOVA, followed by Tukey's multiple comparisons test. *N* = 4. (L, M) The ratio of Ki67^+^ and Nestin^+^ in cerebral organoids with FGFR1 inhibitor (PD173074) pretreatment. Data are mean ± SEM.***p < 0.001, one‐way ANOVA, followed by Tukey's multiple comparisons test. *N* = 4. (N) Representive images of Sox2^+^ and Tuj1^+^ cells in cerebral organoids after chrysin treatment with or without TrkB and FGFR1 inhibitors pretreatment. Scale bar = 200 μm. (O, P) The ratio of Sox2^+^ and Tuj1^+^ in cerebral organoids with TrkB inhibitor (ANA‐12) pretreatment. Data are mean ± SEM. *p < 0.05, ***p < 0.001, one‐way ANOVA, followed by Tukey's multiple comparisons test. *N* = 4. (Q, R) The ratio of Sox2^+^ and Tuj1^+^ in cerebral organoids with FGFR1 inhibitor (PD173074) pretreatment. Data are mean ± SEM. **p < 0.01, ***p < 0.001, one‐way ANOVA, followed by Tukey's multiple comparisons test. *N* = 4.

Next, we examine whether blocking TrkB and FGFR1 also reduces the effect of chrysin on cerebral organoids. Following a 2‐week treatment with TrkB and FGFR1 inhibitors, we observed that not only TrkB inhibition but also FGFR1 inhibition significantly reduced the effects of chrysin on cerebral organoid expansion (Figure 6A–H). Moreover, simultaneous inhibition of both receptors almost entirely abolished the effects of chrysin (Figure S5A–D). Immunofluorescence analysis of organoids demonstrated that the increased proportion of Ki67^+^ cells following chrysin treatment was significantly diminished upon TrkB and FGFR1 inhibition, indicating that the chrysin‐induced increase in NSC proliferation was attenuated by blocking TrkB and FGFR1 (Figure 6I,J,L). In addition, both blockages of the two receptors also nearly shed the effects of chrysin (Figure S5E,F). Consistently, the ratio of Sox2^+^ and Nestin^+^ NSCs, which had been elevated by chrysin treatment, was also reduced upon TrkB and FGFR1 inhibition and almost abolished while both receptors were inhibited (Figures 6K,M,O,Q and S5G,I). Next, we investigated the expression of the neuron marker Tuj1 using immunostaining. The results revealed that chrysin‐induced neuronal differentiation in cerebral organoids was attenuated due to the inhibition of TrkB and FGFR1 (Figures 6N,P,R and S5H,J). In conclusion, our results suggested that blocking TrkB and FGFR1 also reduces the effect of chrysin on neurogenesis in cerebral organoids.

## 4 DISCUSSION

While there is increasing evidence to suggest that flavonoids affect many biological processes, their influence on neurogenesis and the underlying mechanisms are still largely unknown.^35^ In this study, we employed human NSCs and cerebral organoids to show that the flavonoid chrysin not only stimulated the proliferation and self‐renewal of NSCs but also enhanced neuronal differentiation and outgrowth, indicating that chrysin promoted multiple stages of neurogenesis. Mechanistic studies revealed that chrysin exerted its neurogenic effects by cooperatively activating the neurotrophic receptors TrkB and FGFR1. Moreover, we observed that chrysin effectively increased the expression levels of BDNF and FGF1/8. Taken together, our findings suggest that chrysin may serve as a potential candidate for addressing cognitive decline related to ageing and neurodegenerative diseases by enhancing neurogenesis.

The BDNF/TrkB signalling is a central regulator of neurogenesis and synaptic plasticity in the central system and has a strong correlation with cognitive function.^39^ It plays a pivotal role in ameliorating pathological features in a variety of neurological diseases, particularly depression.^40^ The way BDNF/TrkB signalling is activated, whether transiently or sustained, can have varying effects on neuronal cells, indicating that different activation modes may result in diverse molecular and cellular functions.^41^ Our study revealed that treatment with chrysin not only triggered an immediate activation of TrkB receptors, but also sustained this activation for 30 min after the addition of chrysin. Notably, extended chrysin treatment resulted in an up‐regulation of BDNF expression. Our results imply that chrysin might stimulate both transient and sustained BDNF/TrkB signalling by directly activating the receptor and enhancing BDNF expression.

FGF/FGFR1 signalling also influences the proliferation and differentiation of NSCs and is crucial for maintaining neurogenic potential.^24^ It has been shown that FGF enhances BDNF protein expression both in vitro and in vivo.^42^, ^43^ In the present study, we found that chrysin not only enhanced BDNF/TrkB pathways but also stimulated FGF/FGFR1 signalling. This is noteworthy because compared to the activation of BDNF/TrkB signalling alone, concurrent activation of FGF/FGFR1 signalling might be a more effective method, as it could help preserve the activation state of BDNF/TrkB signalling through the up‐regulation of BDNF expression. Furthermore, it has been reported that TrkB can form functional heterodimers with other receptor tyrosine kinases like Her2.^44^ This raises the possibility that TrkB and FGFR1 could similarly engage in heterodimerization, allowing for their simultaneous activation by chrysin. Together, the cooperative activation of TrkB and FGFR1 by chrysin could explain its ability to regulate different stages of neurogenesis. This dual activation mechanism allows chrysin to function efficiently in different physiological and pathological environments.

The therapeutic potential of BDNF and FGF is limited by their poor pharmacokinetics and potential side effects.^41^, ^45^ Our research has identified the flavonoid chrysin, which could serve as a promising agent with the capacity to mimic the neurotrophic functions of these growth factors. In agreement with our observations, chrysin is known to protect against memory deficits induced by the neurotoxic agent methotrexate.^46^ Moreover, chrysin has been shown to ameliorate loss of cognition and hippocampal neurogenesis in the hippocampus in a D‐galactose‐induced ageing rat model.^47^ Despite structural similarities with chrysin, other bioactive flavonoids, including the senolytic drug quercetin, show less effects in neural stem cell proliferation. Our findings suggest two key functionalities of bioactive flavonoids: their senolytic effect and their ability to enhance neurogenesis. Both are crucial in mitigating the cognitive decline linked to ageing and neurodegenerative disorders, as removing senescent cells provides a supportive microenvironment for neurogenesis. Thus, developing innovative agents that merge the therapeutic benefits of various flavonoids, including quercetin and chrysin, offers innovative strategies for combating ageing and neurodegenerative disease.

## AUTHOR CONTRIBUTIONS

**Xiaoxu Dong**: collection and/or assembly of data; data analysis and interpretation; manuscript writing; final approval of manuscript. **Gang Pei**: conception and design; financial support; manuscript writing; final approval of manuscript. **Zhuo Yang**: technical help. **Shichao Huang**: manuscript writing; final approval of manuscript.

## CONFLICT OF INTEREST STATEMENT

The authors declare no conflicts of interest.

## Supporting information


**Data S1.** Supporting Information.

## Data Availability

The data that support the findings of this study are available from the corresponding author upon reasonable request.
