# Supplementary material for: Flavonoid chrysin activates both TrkB and FGFR1 receptors while upregulates their endogenous ligands such as brain derived neurotrophic factor to promote human neurogenesis
Source: Cell Prolif. 2024 Sep 27;58(1):e13732. doi: 10.1111/cpr.13732 (PMC11693565; doi:10.1111/cpr.13732)

SUPPLEMENTARY FIGURES

SUPPLEMENTARY FIGURE 1 Screening of flavonoids and effects of chrysin. (A) Screening of effects of flavonoids on neural stem cell proliferation. (B) Detection of effects of flavonoids on the neuron outgrowth. (C) Time lapses of cell growth in response to chrysin. Data are mean ± SEM. ***p < 0.001, One-way ANOVA, followed by Tukey's multiple comparisons test. N = 4. (D) Dosage-dependent response of cell growth after treatment with chrysin in the other cell line. Data are mean ± SEM. ***p < 0.001, One-way ANOVA, followed by Tukey's multiple comparisons test. N = 3. (E) The number of different diameters secondary neurospheres.

Related to Figure 1.

SUPPLEMENTARY FIGURE 2 Effects of chrysin on astrocytes differentiation and outgrowth of neurites on SHSY-5Y. (A)-(D) Quantitative real-time PCR analysis of Sox2, Nestin and DCX, Tuj1 mRNA levels in vehicle- or chrysin-treated human NSCs. Data were normalized to control. Data are mean ± SEM. ** p < 0.01, ***p < 0.001, unpaired Student's t-test. N = 4. (E) Representative images of immunostaining for GFAP+ and Sox2+ in vehicle- or chrysin-treated human NSCs. Scale bar = 200 µm. (F) Quantification of the co-immunostaining of GFAP+ and Sox2- cells with or without chrysin treatment. Data are mean ± SEM. *p < 0.05, unpaired Student's t-test. N = 3. (G) Immunostaining of the Sox2/GFAP in vehicle- or chrysin-treated human NSC derived astrocytes. Scale bar = 200 µm. (H) Quantification of the co-immunostaining of GFAP+ and Sox2- cells with or without chrysin treatment. Data are mean ± SEM. *p < 0.05, unpaired Student's t-test. N = 3. (I) Representive images of SHSY-5Y with or without chrysin treatment. Scale bar = 100 µm. (J) The neurite length of SHSY-5Y after chrysin treatment and RA used as the positive control. Data are mean ± SEM. ***p < 0.001, one-way ANOVA, followed by Tukey's multiple comparisons test. N = 5. (K) Quantitative real-time PCR analysis of Tuj 1 mRNA levels. Data are mean ± SEM. ** p < 0.01, unpaired Student's t-test. N = 4.

Related to Figure 2.

SUPPLEMENTARY FIGURE 3 Neurotrophic factor receptor TrkB but not TrkA influenced the effects of chrysin. (A) Expression of Trks in human NSC. N = 3. (B) The effects of chrysin on NSC proliferation in the presence or absence of TrkA inhibitor (AG-879) pretreatment. Data are mean ± SEM. ***$p < 0.001$, ns, no significance, one-way ANOVA, followed by Tukey's multiple comparisons test. N = 3. (C-D) The efficacy identification of TrkA and TrkB knock down detected by quantitative real-time PCR analysis. Data are mean ± SEM. *$p < 0.05$, unpaired Student's t-test. N = 3. (E) The effect of chrysin on human NSC growth with TrkA knock down. Data are mean ± SEM. ***$p < 0.001$, ns, no significance, one-way ANOVA, followed by Tukey's multiple comparisons test. N = 3. (F) Time curve of p-TrkB (Y816) response to chrysin treatment. (G) Binding affinity and protein-ligand interactions of TrkB receptors with chrysin on 3D representation and 2D representation. Data were obtained using the software-Maestro. (H-I) The mRNA expression of BDNF and NGF with chrysin treatment in human NSCs. Data are mean ± SEM. ** $p < 0.01$, unpaired Student's t-test. N = 3.
Related to Figure 3.

SUPPLEMENTARY FIGURE 4 Blockage both of TrkB and FGFR1 almost block the effects of chrysin on human NSCs but not on neuron. (A-B) The effects of chrysin on NSC proliferation with inhibitors of VEGFR2 (ZM323881) and EGFR (PKI-166) pretreatment. Data are mean ± SEM. ***$p < 0.001$, ns, no significance, one-way ANOVA, followed by Tukey's multiple comparisons test. N = 3. (C) Expression of FGFR1, FGFR2, FGFR3, FGFR4 in human NSCs. N = 3. (D) Binding affinity and protein-ligand interactions of FGFR1 receptors with chrysin on 3D representation and 2D representation. Data were obtained using the software-Maestro. (E-G) The mRNA expression of FGF1/2/3/4/8 and EGF with chrysin treatment in human NSCs. Data are mean ± SEM. *$p < 0.05$, ***$p < 0.001$, unpaired Student's t-test. N = 3. (K) Proliferation of NSCs with chrysin treatment in the presence or

absence of co-inhibition of TrkB and FGFR1. Data are mean ± SEM. ** $p < 0.01$, one-way ANOVA, followed by Tukey's multiple comparisons test. N = 3. (L-N) The formation of secondary neurospheres after chrysin treatment with TrkB and FGFR1 inhibitors co-pretreatment. Data are mean ± SEM. *$p < 0.05$, ***$p < 0.001$, one-way ANOVA, followed by Tukey's multiple comparisons test. N = 4. (O) The neurite length on SHSY-5Y with chrysin treatment in presence of different inhibitors pretreatments. Data are mean ± SEM. *$p < 0.05$, ***$p < 0.001$, one-way ANOVA, followed by Tukey's multiple comparisons test. N = 3.
Related to Figure 4.

SUPPLEMETARY FIGURE 5 Inhibition of TrkB and FGFR1 simultaneously diminished the effects of chrysin in cerebral organoids. (A-D) Light sheet images and reconstructed models of control and chrysin-treated organoids with or without TrkB and FGFR1 overlaying inhibition treatment and quantification of volume, surface area, and sphericity at 2 weeks (n = 3 cerebral organoids per condition). Scale bar = 200 μm. Data are mean ± SEM. **$p < 0.01$, ***$p < 0.001$, one-way ANOVA, followed by Tukey's multiple comparisons test. N = 4. (E) Representative images of $Ki67^+$ and $Nestin^+$ cells in cerebral organoids with or without TrkB and FGFR1 overlaying inhibition treatment at 3 weeks. Scale bar = 200 μm. (F-G) Quantification of $Ki67^+$ and $Nestin^+$ cells to $DAPI^+$ cell in different groups. Data are mean ± SEM. ***$p < 0.001$, one-way ANOVA, followed by Tukey's multiple comparisons test. N = 4. (H) Representative images of $Sox2^+$ and $Tuj1^+$ in cerebral organoids with or without TrkB and FGFR1 overlaying inhibition treatment at 3 weeks. Scale bar = 200 μm. (I-J) Quantification of $Sox2^+$ and $Tuj1^+$ cells to $DAPI^+$ cell in different groups. Data are mean ± SEM. **$p < 0.01$, ***$p < 0.001$, one-way ANOVA, followed by Tukey's multiple comparisons test. N = 4.
Related to Figure 6.

SUPPLEMENTARY FIGURE 1 Screening of flavonoids and effects of Chrysin.

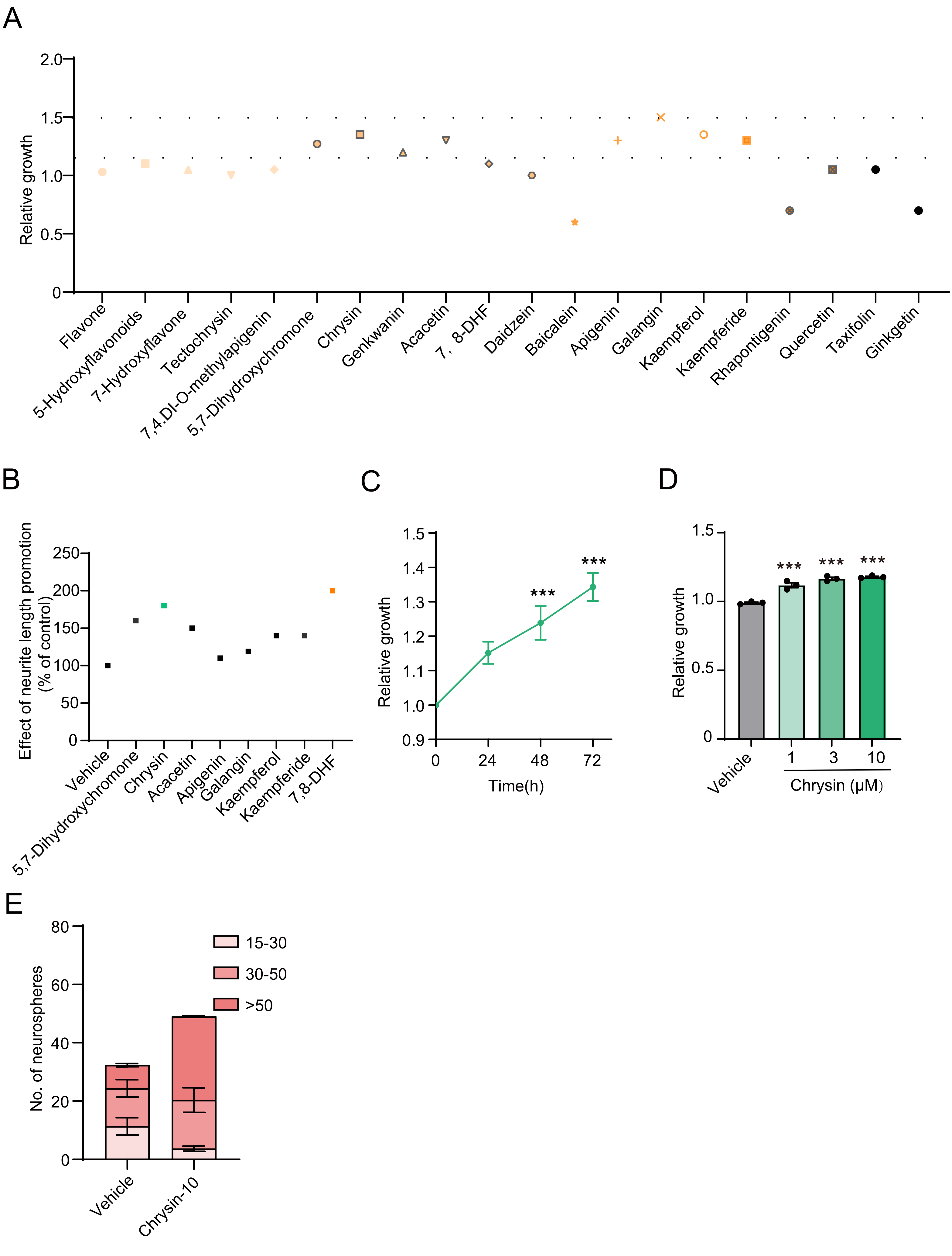

SUPPLEMENTARY FIGURE 2 Effects of Chrysin on the astrocytes differentiation and outgrowth of neurites on SHSY-5Y cells.

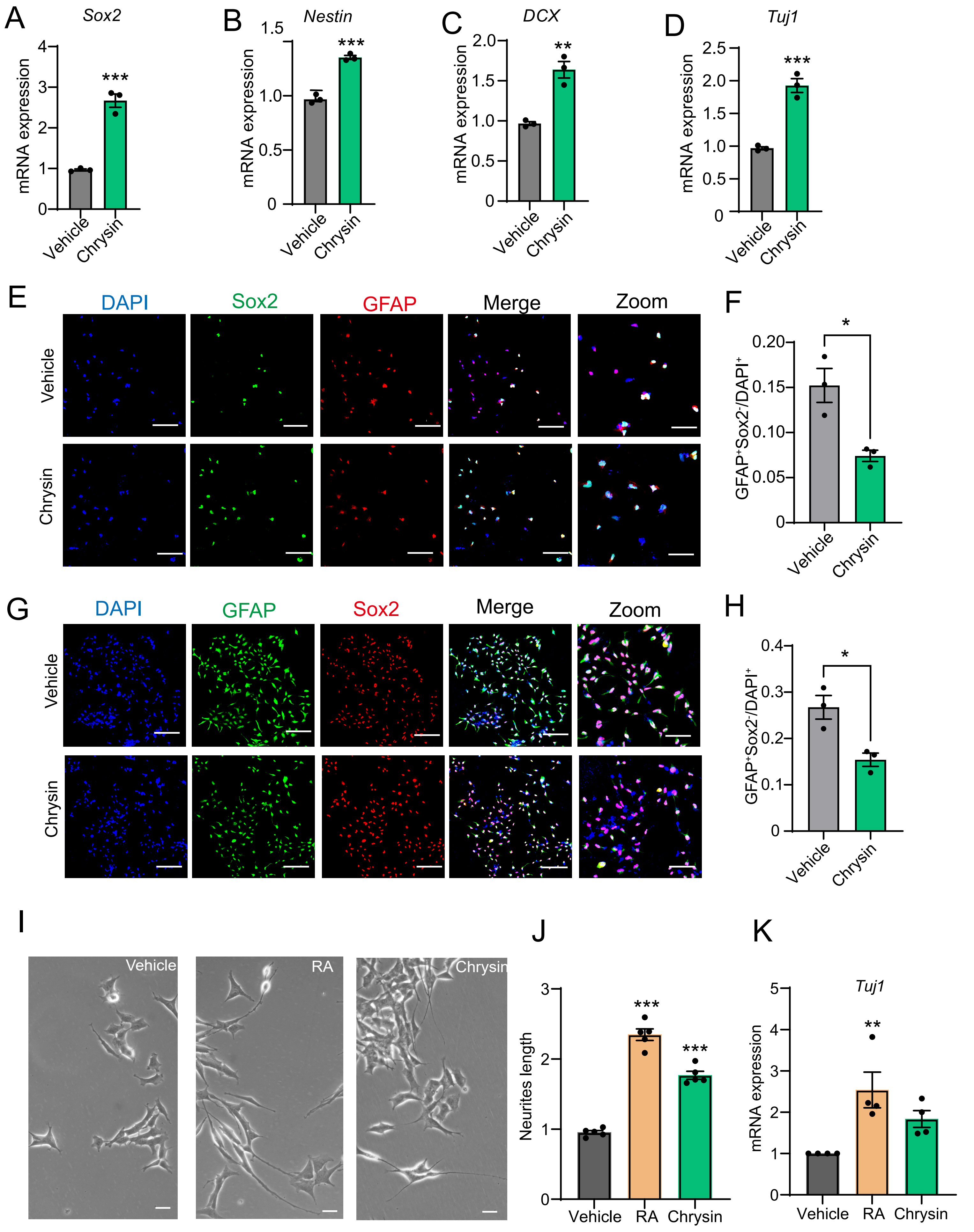

SUPPLEMENTARY FIGURE 3 Neurotrophic factor receptor TrkB but not TrkA influenced the effects of chrysin.

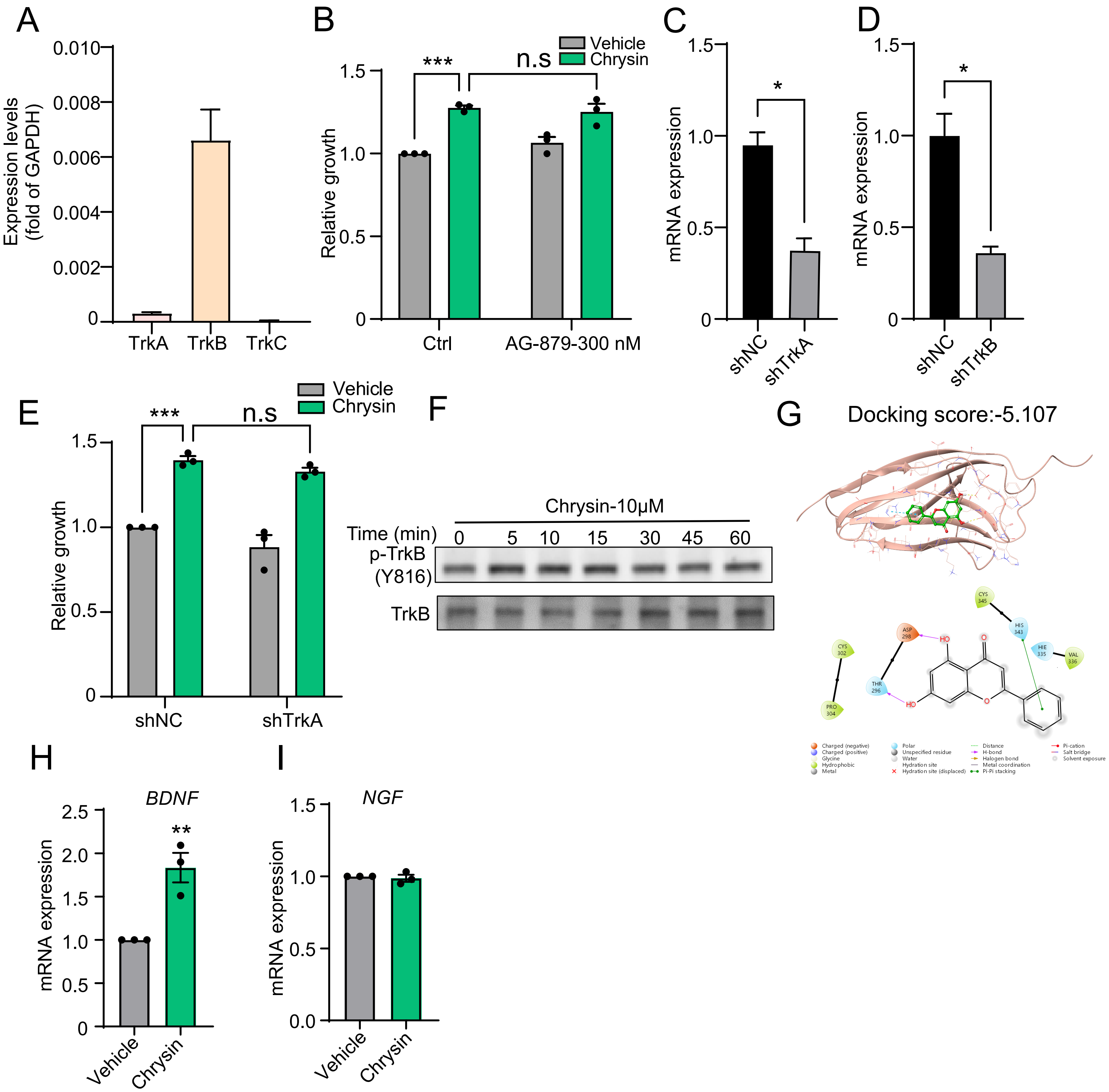

SUPPLEMENTARY FIGURE 4 Blockage both of TrkB and FGFR1 almost block the effects of chrysin on human NSCs but not on neuron.

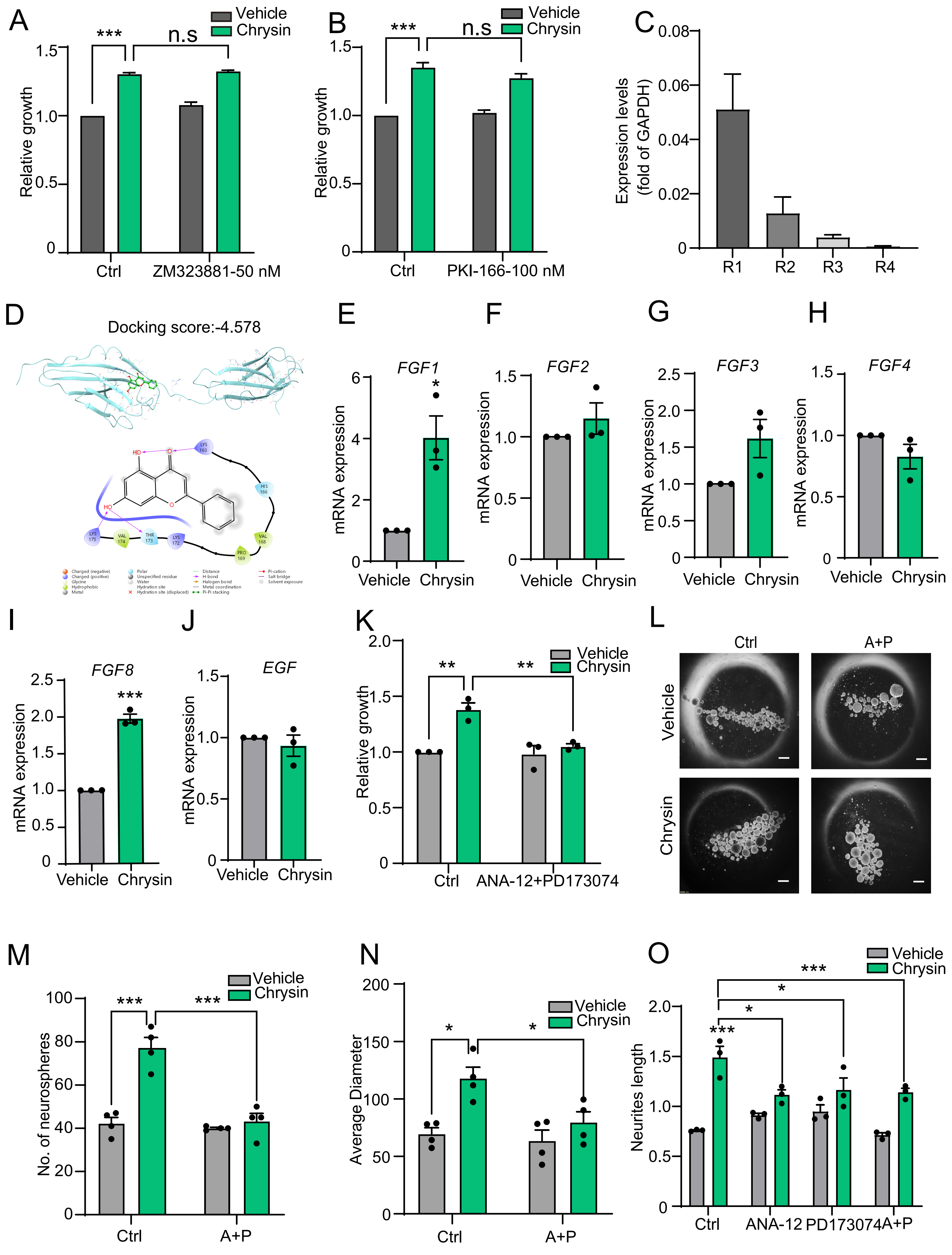

SUPPLEMENTARY FIGURE 5 Inhibition of TrkB and FGFR1 simultaneously diminished the effects of chrysin in cerebral organoids.

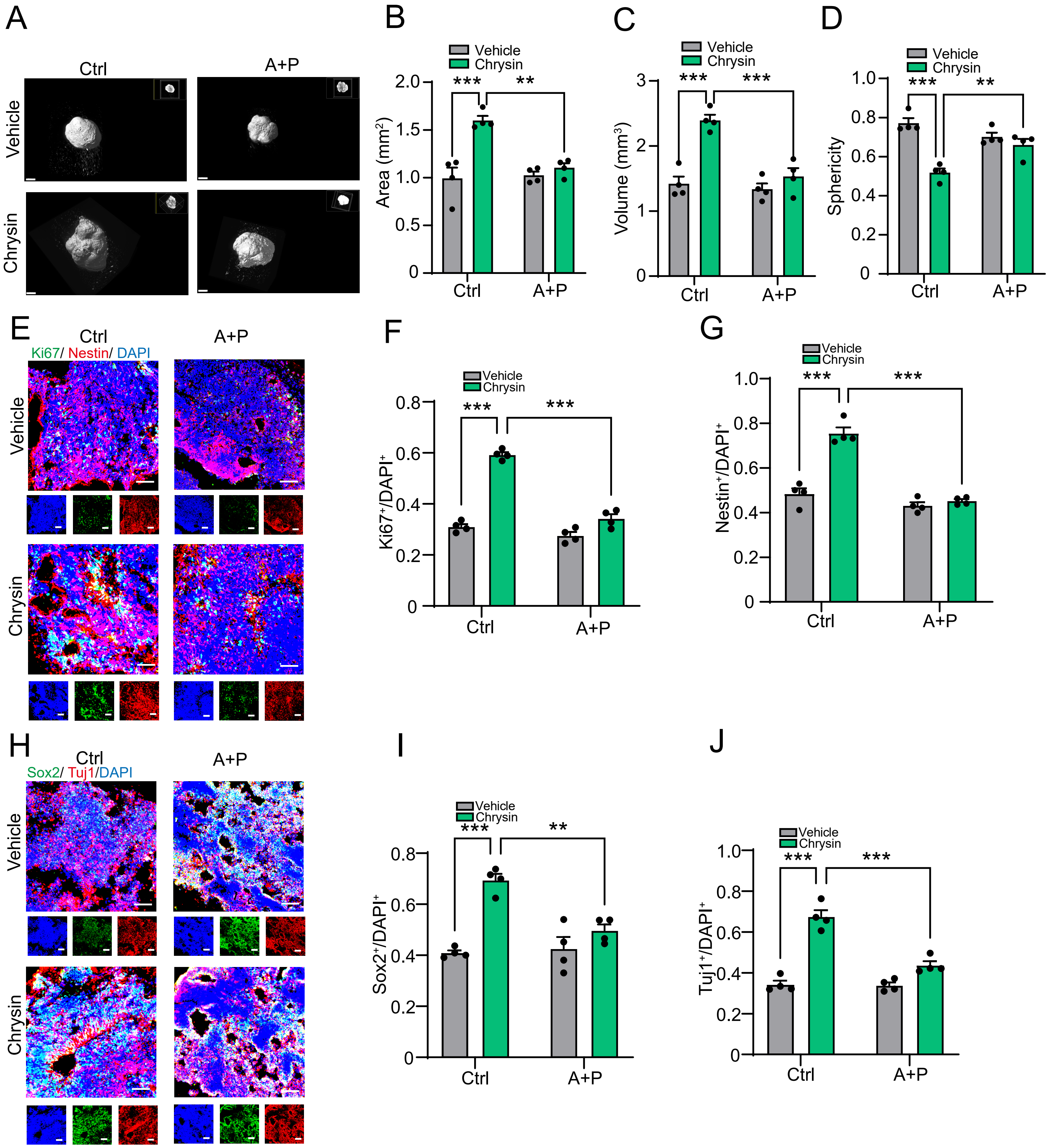

Supplement: Supplementary file 1 — Data S1. Supporting Information. [file CPR-58-e13732-s001.pdf]
